# microRNA‐651‐5p affects the proliferation, migration, and invasion of lung cancer cells by regulating Calmodulin 2 expression

**DOI:** 10.1111/crj.13665

**Published:** 2023-07-20

**Authors:** Yaoguo Lang, Xianglong Kong, Benkun Liu, Xiangyuan Jin, Lantao Chen, Shidong Xu

**Affiliations:** ^1^ Department of Thoracic Surgery Harbin Medical University Cancer Hospital Harbin Heilongjiang China

**Keywords:** apoptosis, CALM2, invasion, lung cancer, microRNA‐651‐5p, migration, proliferation

## Abstract

**Objective:**

Lung cancer is prevalent worldwide and a leading contributor to tumor death. This research intends to explore the molecular mechanism of the microRNA‐651‐5p (miR‐651‐5p)/Calmodulin 2 (CALM2) axis in the proliferation, migration, and invasion of lung cancer cells.

**Methods:**

Lung cancer tissues and para‐cancerous tissues were collected. The expression levels of miR‐651‐5p and CALM2 in lung cancer tissues and cells were tested, and the connection between miR‐651‐5p expression and clinicopathological characteristics of lung cancer patients was further analyzed. The binding sites between miR‐651‐5p and CALM2 were analyzed and validated. Lung cancer cell proliferation, migration, invasion, and apoptosis were examined.

**Results:**

miR‐651‐5p was lowly expressed in lung cancer tissues and cells. miR‐651‐5p expression had no correlation with patients' age and gender but had a correlation with patients' tumor size, TNM stage, and lymph node metastasis. Overexpression of miR‐651‐5p repressed proliferative, migratory, and invasive behaviors of lung cancer cells. miR‐651‐5p targeted and negatively regulated CALM2 expression, and CALM2 reversed the inhibiting effects of miR‐651‐5p on lung cancer cell malignant behaviors, including proliferation, migration, and invasion.

**Conclusion:**

This study expounds that miR‐651‐5p affects the proliferation, migration, and invasion of lung cancer cells by regulating CALM2 expression.

## Graphical abstract

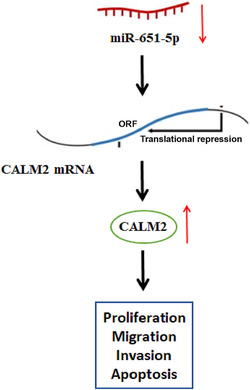

## 1 INTRODUCTION

Lung cancer is prevalent all over the world and the leading contributor to tumor death, of which non‐small cell lung cancer (NSCLC) accounts for 80%–85%.^1^ The main elements linked to the occurrence of lung cancer are tobacco smoking, radon exposure, domestic fuel smoke, infection, and human papilloma virus.^2^ Early detection of lung cancer can significantly improve 5‐year survival, from less than 5% in stage IV to 54%–73% in stage I.^3^ However, in spite of the improvements in screening, prevention, and treatment, 5‐year survival rates of patients with lung cancer remain low.^4^ Thus, the requirements for novel therapeutic options are in emergency.

microRNAs (miRNAs) are a group of endogenous non‐coding RNAs that modulate the expression levels of genes.^5^ miRNA acts as either a tumor‐promoter or an inhibitor depending on the tumor types.^6^ miRNAs are good candidates as biomarkers for lung cancer.^7^ For example, miR‐651‐5p expression is associated with sebaceous gland carcinoma (SGC) malignant behaviors.^8^ It is also suggested that miR‐651‐5p expression has an association with colorectal cancer (CRC).^9^ A previous study has demonstrated that miR‐651‐5p‐mediated oncogenicity manipulation in hepatocellular carcinoma (HCC) may provide guiding significance on the diagnosis and therapy for HCC.^10^ Calmodulin 2 (CALM2) is one of the encoding identical calmodulin proteins.^11^ It serves as one member of the CALM family, and its upregulation confers resistance to afatinib in gastric cancer (GC) cells.^12^ Targeting CALM2 might be a molecular method in treating primary HCC and preventing metastasis or recurrence.^13^ CALM2 can function in facilitating GC metastasis and angiogenesis.^14^ In addition, it is reported that there is a targeting relationship between CALM2 and miRNAs.^15^ For example, miR‐338‐3p can directly target CALM2 to repress oxygen‐glucose deprivation (OGD)‐induced damage.^16^ In general, the independent function of miR‐651‐5p and CALM2, together with their multilateral interplay in lung cancer, has not been extensively explored. Given the complexity of the interplay among miR‐651‐5p and CALM2, and the knowledge vacancy, there is a pressing requirement to translate the mechanism of these two factors in lung cancer. Therefore, this research intends to explore the molecular mechanism of the miR‐651‐5p/CALM2 axis in the malignant behaviors of lung cancer cells.

## 2 MATERIALS AND METHODS

### 2.1 Clinical samples

Clinical specimens were collected from 68 pairs of NSCLC tissues and non‐tumor tissues of patients who underwent surgery in Harbin Medical University Cancer Hospital. All cases had not received chemotherapy, radiotherapy, or any other special treatments before sampling. All the sections were confirmed by two experienced pathologists. The ages of patients ranged from 42 to 73 years, and the mean age was 57 years. The obtained tissues were grouped into two parts, one immediately preserved in liquid nitrogen while the other fixed in 10% formaldehyde and then embedded in paraffin.^17^

### 2.2 Cell culture and transfection

Lung cancer cell lines (H1299, A549, H358, and H522) and non‐neoplastic bronchial epithelial cells BEAS‐2B were obtained from the Institute of Biochemistry and Cell Biology, Shanghai Institutes for Biological Sciences, Chinese Academy of Sciences. Lung cancer cell lines were incubated in Dulbecco's modified eagle's medium (DMEM) (Gibco, Grand Island, NY, USA) containing 10% fetal bovine serum (FBS) (Gibco), 100 U/mL penicillin and 100 μg/mL streptomycin (Gibco), while BEAS‐2B cells were incubated in LHC‐9 complete medium (Gibco), both cultured at 37°C under 5% CO_2_. miR‐651‐5p and CALM2 overexpression sequences and control sequences as well as CALM2 interference sequences and control sequences were designed, and the synthesized transfection sequences were obtained from Sangon Biotech (Shanghai, China). Lipofectamine 2000 reagent (Invitrogen, Carlsbad, USA) was utilized to transfect lung cancer cells. After transfection, the cells in different groups were incubated at 37°C and 5% CO_2_ for 48 h for further experiments.^18^

### 2.3 Reverse transcription quantitative polymerase chain reaction (RT‐qPCR)

Total RNA in tissues and cells was isolated utilizing Trizol kit (Sigma‐Aldrich, SF, CA, USA), and RNA concentration and purity were measured. Relative to the reverse transcription kit (Fermentas, Maryland, NY, USA) specifications, RNA reverse transcription into complementary DNA was performed, which was then stored at −20°C. The primer sequences of miR‐651‐5p and CALM2 are listed in Table S1. RT‐qPCR was performed on a real‐time PCR kit (Takara, Dalian, China) and detected by real‐time PCR (ABI 7500, ABI, Foster City, CA, USA). U6 was adopted as the internal control for miR‐651‐5p and glyceraldehyde‐3‐phosphate dehydrogenase for CALM2. The 2^‐ΔΔCt^ method was conducted to evaluate the relative expression of target genes.

### 2.4 Western blot analysis

The tissues were taken, placed in a centrifuge tube, followed an addition of 100 μL of radio immunoprecipitation assay (RIPA) lysis solution (Solarbio, Beijing, China) (containing 1 mmol/L phenylmethanesulfonyl fluoride [PMSF], supplemented as needed) and then homogenized at 3000 r/min until fully lysed. Next, the mixture was put on ice for 4 min at 4°C and centrifuged at 12000 *g* for 4 min. Following, the supernatants were isolated, divided into portions and preserved at −80°C. According to the instructions of the bicinchoninic acid assay (BCA) kit (Boster, Wuhan, China), the protein concentration was determined, with the concentration of each sample adjusting to 3 μg/μL. The extracted protein was boiled at 95°C for 10 min after supplementing with loading buffer. Then the proteins were separated with 30 μg loading buffer and 10% polyacrylamide gel electrophoresis per well. The proteins were transferred to polyvinylidene difluoride (PVDF) membrane (Sigma, USA) by semi‐dry electrotransfer method, sealed with 5% bovine serum albumin (BSA) (Beijing Biopartner Science & Technology Co., Ltd., China) at room temperature for 1 h, followed an addition of rabbit anti‐CALM2 (1:5000; Abcam, UK), and incubated at 4°C overnight. After rinsing, the membrane was then supplemented with the corresponding goat anti‐rabbit secondary antibody (1:2000, Abcam, USA) and incubated for 1 h at room temperature. After that, chemiluminescence reagent was implemented to develop the membrane, with GAPDH (1:5000; Proteintech) as the internal reference. To develop the bands, Bio‐rad Gel Dol EZ imager (GEL DOC EZ IMAGER, Bio‐rad, California, USA) was implemented. The target bands were analyzed utilizing Image J software for gray‐scale values.

### 2.5 Cell counting kit‐8 (CCK‐8) assay

After transfection for 48 h, the cells were inoculated in 96‐well culture plates for 3 h, with 1 × 10^3^ cells per well. Incubation for 24, 48, and 72 h later, 100 μL of fresh DMEM without FBS or penicillin–streptomycin solution and 10 μL of CCK‐8 solution (Dojindo, Kumamoto, Japan) were supplemented to each well, followed incubation for 1 h at 37°C. The optical density (OD) value at 490 nm was measured utilizing a spectrophotometer (BioTek Instruments, Winooski, VT, USA).^19^

### 2.6 Transwell assay

The Transwell® assay (with or without Matrigel®) was utilized to measure cell migration and invasion. Cell migration assay: The transfected cells of each group (200 μL, 5 × 10^4^ cells/mL) were inoculated in the upper chamber of the transfected well. After 24‐h incubation, the cells migrating to the lower chamber were stained with Giemsa. Migration rate = number of migrated cells/total cells. Cell invasion assay: Matrigel®‐precoated Transwell® chamber (Corning, USA) was utilized, and the transfected cells of each group (200 μL, 5 × 10^4^ cells/mL) were inoculated in the upper chamber of the transwell coated with Matrigel. After 24‐h incubation, the cells retained in the lower half were stained with Giemsa. Invasion rate = number of invading cells/total cells. A DMI4000B microscope (Leica, Wetzlar, Germany) was implemented to obtain images and five fields of view were randomly selected to count cells.^20^, ^21^

### 2.7 Flow cytometry

After transfection for 48 h, the cells were digested with 0.25% trypsin (without ethylenediaminetetraacetic acid [EDTA]) (Shanghai Yu Bo Biotech Co., Ltd., Shanghai, China), preserved in a flow tube, and conducted centrifugation, and then the supernatants were discarded. After that, the cells were centrifuged and the supernatants were discarded. Based on the instructions of the Annexin‐V‐FITC Apoptosis Assay Kit (Biovision, USA), the Annexin‐V‐FITC/PI staining solution was prepared in a 1:2:50 ratio of Annexin‐V‐FITC, PI, and HEPES cache solution. Every 100 μL of dye solution was resuspended with 1 × 10^6^ cells, fully shaking and mixing. After 15‐min incubation at room temperature, 1 mL of HEPES buffer solution (Procell, Wuhan, China) was supplemented and shaken well. To assess apoptosis, FITC and PI fluorescence were measured by excitation of 525 and 620 nm bandpass filters at 488 nm, respectively.^22^

### 2.8 Dual luciferase reporter gene assay

The target site sequence (wild type, WT) of CALM2 mRNA 3’‐UTR region and the sequence after targeted mutation from WT target site (MUT) were synthesized. Restriction endonuclease was implemented to digest the pmiR‐RB‐REPORTTM plasmids (Guangzhou Ruibo Biotechnology Co., Ltd., Guangzhou, China), and then the pmiR‐RB‐REPORTTM vector was inserted with the synthetic target gene fragments WT and MUT, respectively. The correctly sequenced luciferase reporter plasmids WT and MUT were cotransfected into HEK293T cells with mimic NC or miR‐651‐5p mimic, respectively. At 48 h after transfection, the cells were collected, lysed, and centrifuged for 3–5 min. Next, the supernatants were taken. The relative fluorescence values were obtained by measuring the luciferase reaction intensity (RLU1) utilizing the luciferase assay kit (Beyotime, Shanghai, China), respectively, with renilla luciferase being the internal reference (RLU2).

### 2.9 Statistics

SPSS 21.0 software (IBM SPSS Statistics, Chicago, IL, USA) was applied for statistical analysis. Measurement data were presented as mean ± standard deviation. The paired *t*‐tests were utilized to compare data in a paired design conformed to normal distribution and homogeneity of variance between two groups, and the unpaired *t*‐tests were utilized to compare data in an unpaired design conformed to normal distribution and homogeneity of variance between two groups. The correlation between the expression levels of miR‐651‐5p and the clinicopathological characteristics were tested by chi‐square test. One‐way analysis of variance (ANOVA) was utilized to compare data among multiple groups, with Tukey's for post hoc test. Repeated measures ANOVA was implemented to compare data among groups at different time points. *p* < 0.05 was considered of statistical significance.

## 3 RESULTS

### 3.1 miR‐651‐5p is lowly expressed in lung cancer cells and tissues

**FIGURE 1:**
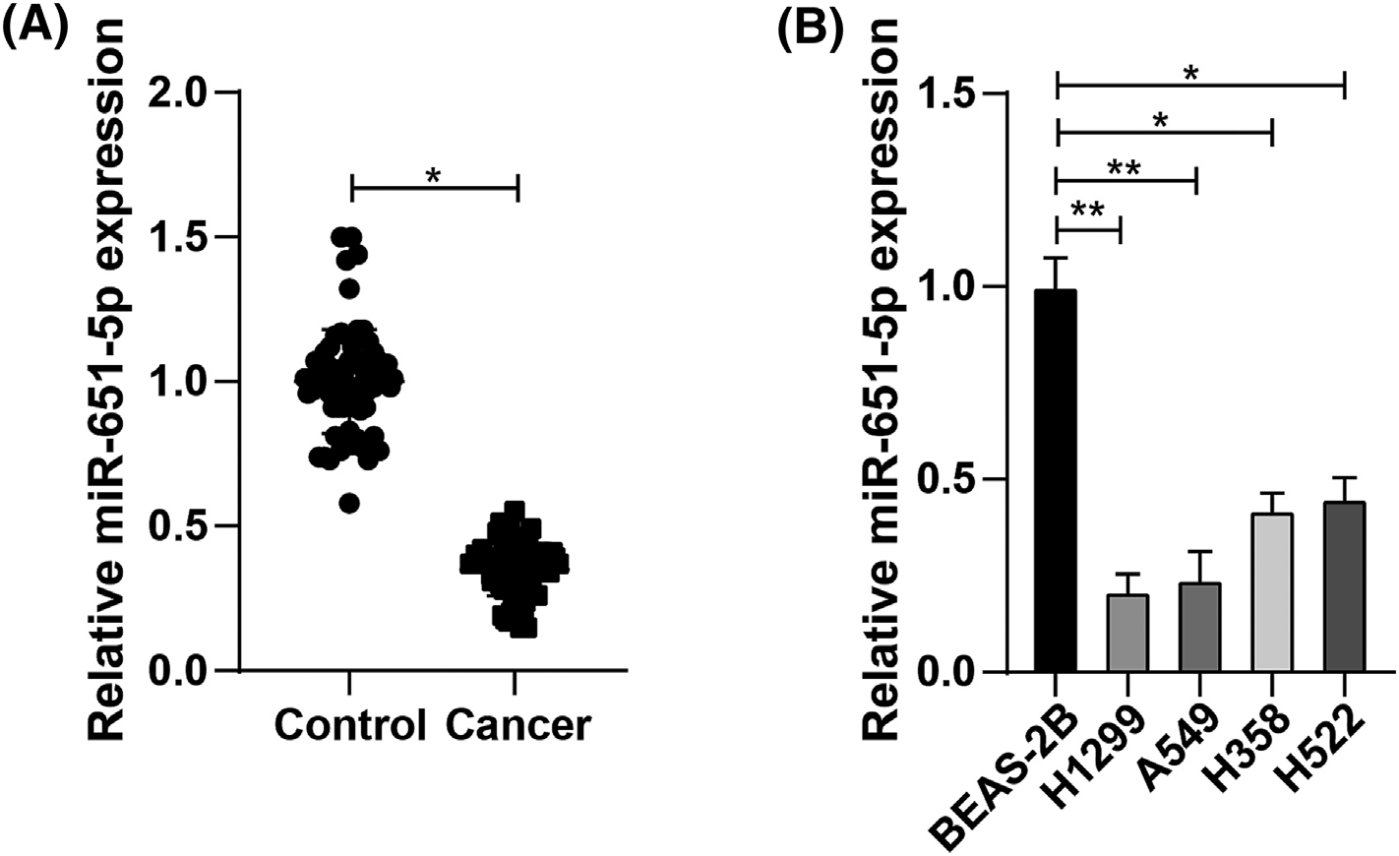
miR‐651‐5p is lowly expressed in lung cancer tissues and cells. (A) miR‐651‐5p expression in tumor tissues was measured by reverse transcription quantitative polymerase chain reaction (RT‐qPCR), *n* = 68; (B) miR‐651‐5p expression in lung cancer cell lines was tested by RT‐qPCR; * *p* < 0.05; ** *p* < 0.01.

As previously reported, overexpression of miR‐651‐5p inhibits tumor progression in colon and liver cancer.^9^, ^10^ However, its expression in lung cancer has not been further investigated. To further verify the connection between miR‐651‐5p and lung cancer, we performed RT‐qPCR and found that miR‐651‐5p was lowly expressed in lung cancer tissues in comparison with that in para‐cancerous tissues (Figure 1A). Versus that in bronchial epithelial cells BEAS‐2B, the expression of miR‐651‐5p in lung cancer cell lines H1299, A549, H358, and H522 was reduced, and the lowest expression was found in H1299 cells, which were selected for following research (Figure 1B).

To investigate the correlation between miR‐651‐5p expression levels and clinicopathological characteristics of lung cancer, lung cancer patients were divided into the high miR‐651‐5p expression group and the low miR‐651‐5p expression group using the median value of miR‐651‐5p expression levels in lung cancer tissues as the threshold. The statistical scores indicated that miR‐651‐5p expression did not correlate with patients' age and gender (*p* > 0.05) but had a correlation with patients' tumor size, TNM stage, and lymph node metastasis (Table S2).

### 3.2 Overexpression of miR‐651‐5p represses the proliferation, migration, and invasion of lung cancer cells and stimulates apoptosis

**FIGURE 2:**
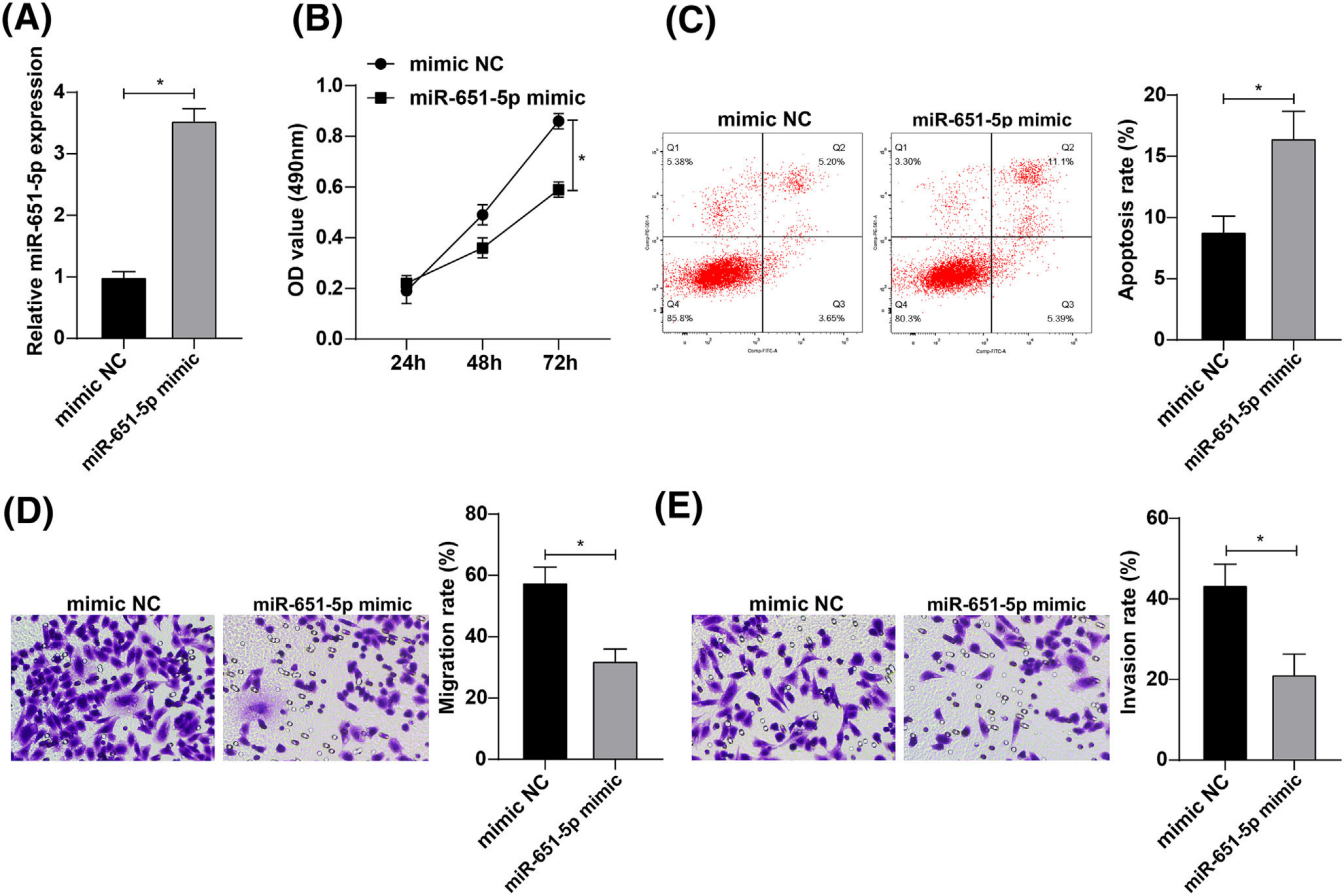
miR‐651‐5p expression affects the biological functions of lung cancer cells. (A) miR‐651‐5p overexpression transfection efficiency was tested by reverse transcription quantitative polymerase chain reaction (RT‐qPCR); (B) The proliferation ability of lung cancer cells after miR‐651‐5p overexpression was assessed by cell counting kit‐8 (CCK‐8) assay; (C) The apoptosis rate after miR‐651‐5p overexpression was tested by flow cytometry; (D–E) The cell migration and invasion ability after miR‐651‐5p overexpression was measured by Transwell assay; * *p* < 0.05.

H1299 cells were treated with miR‐651‐5p overexpression, and RT‐qPCR was conducted to verify the transfection efficiency (Figure 2A). The proliferation and apoptosis capacity of cells in each group was measured by CCK‐8 assay and flow cytometry, respectively, and the experimental results demonstrated that the proliferation ability of cells after overexpressing miR‐651‐5p was reduced and the apoptosis rate was elevated (Figure 2B–C). Transwell assay findings unearthed that cell migration and invasion capacity after overexpressing miR‐651‐5p was decreased (Figure 2D–E). The above results indicated that miR‐651‐5p overexpression suppressed lung cancer cell proliferation, migration, and invasion and promoted apoptosis.

### 3.3 miR‐651‐5p targets and negatively modulates CALM2 expression

**FIGURE 3:**
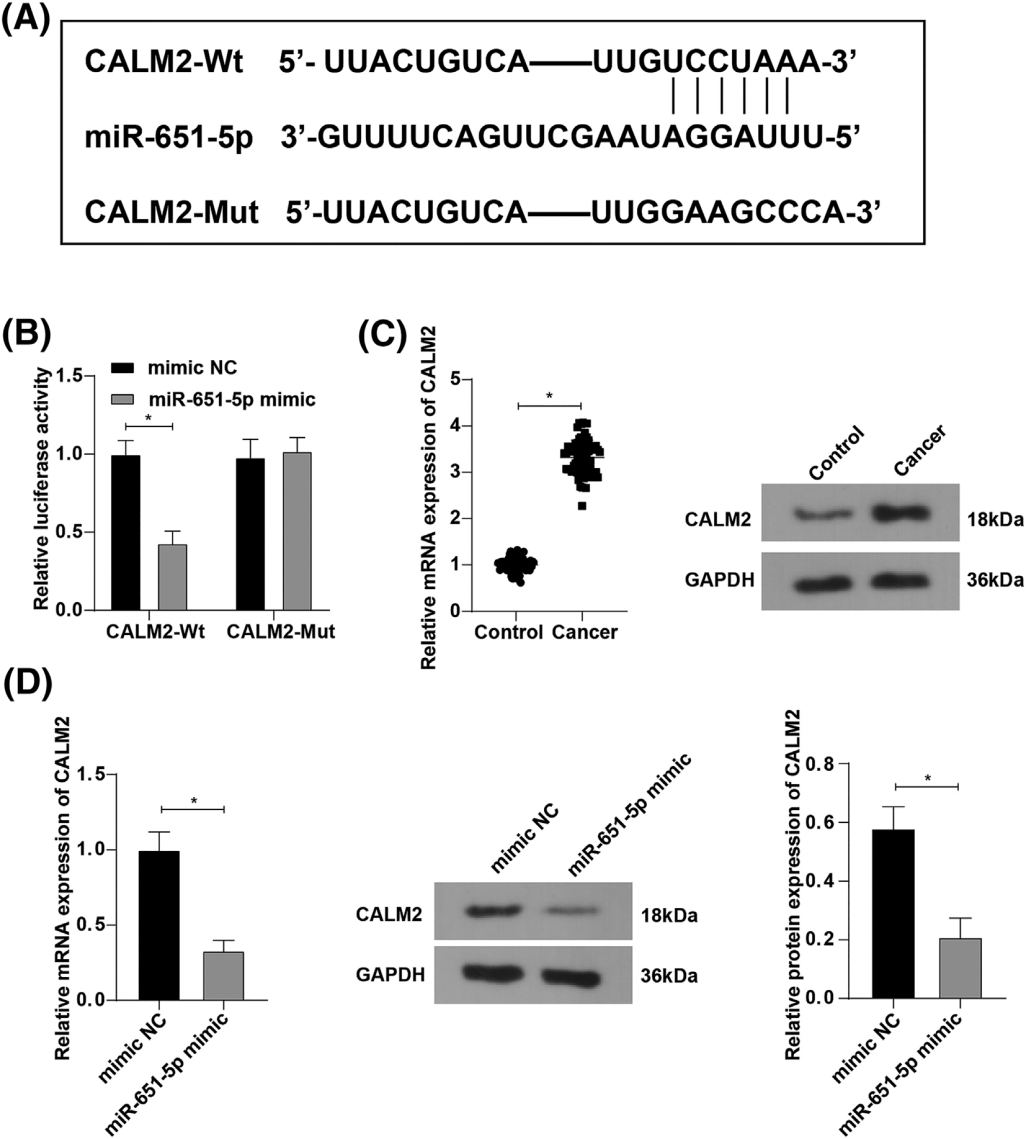
Calmodulin 2 (CALM2) is a target gene of miR‐651‐5p. (A) The target gene of miR‐651‐5p was predicted by bioinformatics websites; (B) The targeting relationship between CALM2 and miR‐651‐5p was verified by dual luciferase reporter gene assay; (C) CALM2 expression in tumor tissues was assessed by reverse transcription quantitative polymerase chain reaction (RT‐qPCR) and western blot assay, *n* = 68; D: CALM2 expression in H1299 cells after miR‐651‐5p overexpression was tested by RT‐qPCR and western blot assay; * *p* < 0.05.

The starbase website (https://starbase.sysu.edu.cn/) predicted the target gene of miR‐651‐5p and found that miR‐651‐5p possessed a target binding site with CALM2 (Figure 3A). It is reported that CALM2 is involved in poor prognosis in lung cancer patients.^23^ Dual luciferase reporter gene assay findings (Figure 3B) unraveled that miR‐651‐5p specifically bound to CALM2, and CALM2 was a target gene of miR‐651‐5p. RT‐qPCR and western blot assay were conducted to test CALM2 expression in lung cancer tissues, and the experimental results indicated that CALM2 expression was elevated in lung cancer tissues compared with that in para‐cancerous tissues (Figure 3C). CALM2 expression in H1299 cells after overexpressing miR‐651‐5p was reduced (Figure 3D). The above findings unearthed that miR‐651‐5p targeted and negatively modulated CALM2 expression.

### 3.4 Interfering CALM2 inhibits lung cancer cell proliferation, migration, and invasion and promotes apoptosis

**FIGURE 4:**
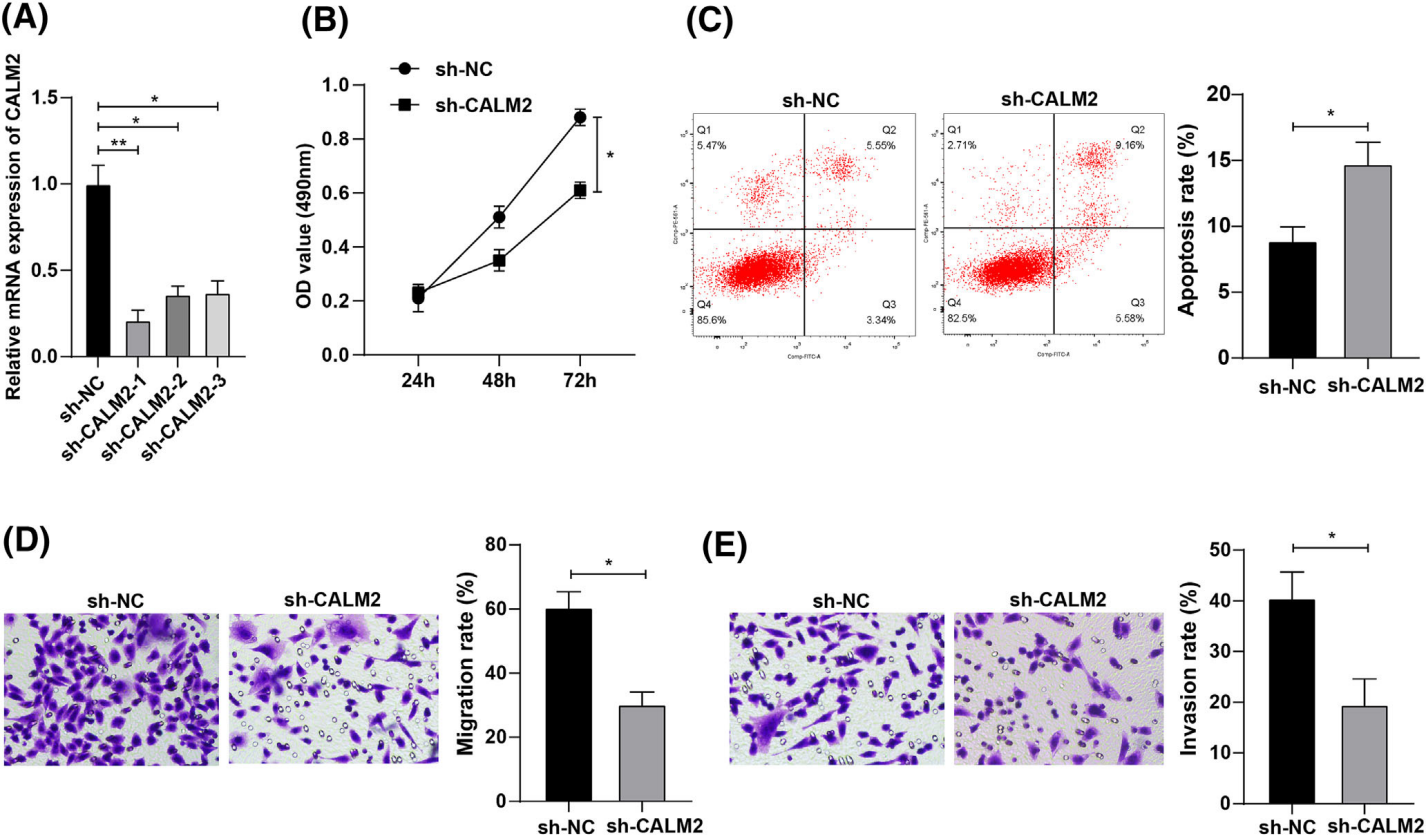
Calmodulin 2 (CALM2) expression affects the biological functions of lung cancer cells. (A) CALM2 interference transfection efficiency in H1299 cells was tested by reverse transcription quantitative polymerase chain reaction (RT‐qPCR); (B) The proliferation ability of lung cancer cells after CALM2 interference was measured by cell counting kit‐8 (CCK‐8) assay; (C) The apoptosis rate after CALM2 interference was assessed by flow cytometry; (D–E) The cell migration and invasion ability after CALM2 interference was determined by Transwell assay; * *p* < 0.05.

To further investigate the effects of CALM2 expression on lung cancer cell biological functions, H1299 cells were treated with CALM2 interference and RT‐qPCR was performed to verify the transfection efficiency (Figure 4A). The proliferation and apoptosis capacity of cells in each group were measured by CCK‐8 assay and flow cytometry, and the results unraveled that after CALM2 interference, the cell proliferation ability was reduced and the apoptosis rate was elevated (Figure 4B–C). The result of Transwell assays demonstrated that the cell migratory and invasive ability after CALM2 interference was reduced (Figure 4D–E). The above findings unraveled that interfering CALM2 repressed the proliferative, migratory, and invasive behaviors of lung cancer cells and promoted apoptosis.

### 3.5 CALM2 reverses the inhibitory effects of miR‐651‐5p on lung cancer cell proliferation, migration, and invasion

**FIGURE 5:**
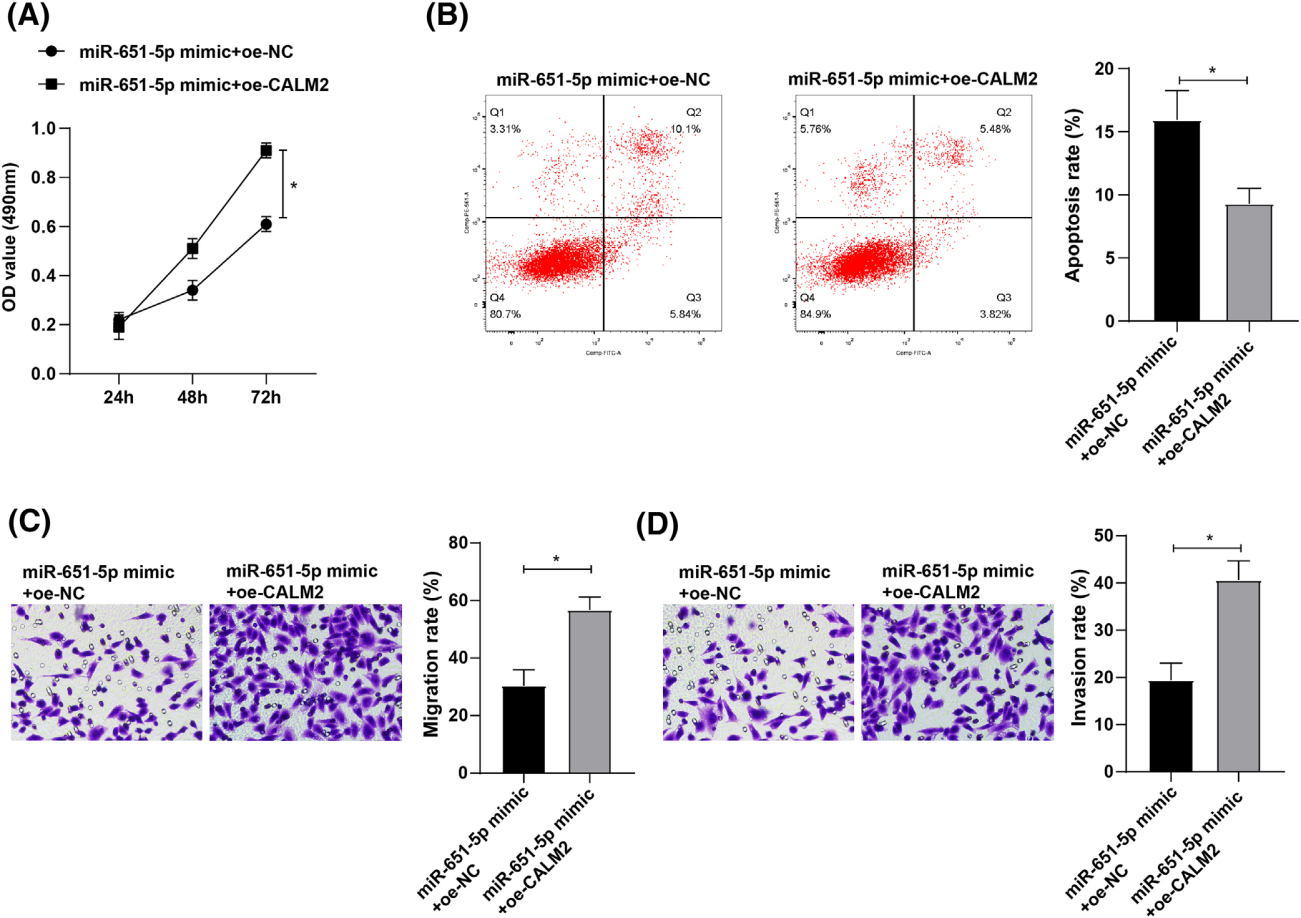
miR‐651‐5p affects the biological functions of lung cancer cells by regulating calmodulin 2 (CALM2) expression. (A) The proliferation ability of lung cancer cells after miR‐651‐5p and CALM2 overexpression was detected by cell counting kit‐8 (CCK‐8) assay; (B) The apoptosis rate after miR‐651‐5p and CALM2 overexpression was measured by flow cytometry; (C–D) The cell migration and invasion ability after miR‐651‐5p and CALM2 overexpression was determined by Transwell assay; * *p* < 0.05.

To further investigate whether miR‐651‐5p affects lung cancer cell functions through CALM2, we set up the miR‐651‐5p mimic + oe‐NC group and the miR‐651‐5p mimic + oe‐CALM2 group for H1299 cells. The proliferation and apoptosis of cells in each group were tested by CCK‐8 assay and flow cytometry, respectively. The experimental results revealed that versus that in the miR‐651‐5p mimic + oe‐NC group, the cell proliferation ability was increased and the apoptosis rate was decreased in the miR‐651‐5p mimic + oe‐CALM2 group (Figure 5A–B). Transwell assay results unearthed that versus that of the miR‐651‐5p mimic + oe‐NC group, the migratory and invasive abilities were impeded in the miR‐651‐5p mimic + oe‐CALM2 group (Figure 5C–D). These results suggested that CALM2 reversed the inhibitory effects of miR‐651‐5p on lung cancer cell proliferative, migratory, and invasive behaviors.

## 4 DISCUSSION

Lung cancer is a malignancy overwhelmed in men and the major reason of death in male and female in most countries.^24^ NSCLC represents 85% of lung tumors.^25^ NSCLC that harbors specific genetic alterations could be pretty sensitive to targeted therapies.^26^ However, the exploration of the internal connections between miR‐651‐5p and CALM2 is in its infancy. Thus, this study is to disinter the mechanism of miR‐651‐5p/CALM2 axis in lung cancer with the findings exhibiting that miR‐651‐5p affects lung cancer cell proliferative, migratory, and invasive behaviors through the regulation of CALM2 expression.

At the outset, we have explored miR‐651‐5p and CALM2 expression in lung cancer tissues and cells and the connection between clinicopathological characteristics and poorer prognosis of patients with lung cancer with miR‐651‐5p expression. It is manifested that miR‐651‐5p is lowly expressed in lung cancer tissues and cells and miR‐651‐5p expression was correlated with patients' tumor size, TNM stage, and lymph node metastasis. As previously reported, miR‐651‐5p overexpression has the ability to inhibit tumor progression in CRC and HCC. In addition, miR‐651‐5p is lowly expressed in CRC and HCC cells and tissues.^9^, ^10^ Also, a study has reported that overexpression of miR‐651‐5p suppresses SGC cell malignant actions. More specifically, miR‐651‐5p not only promotes cell apoptosis but also inhibits the invasive and migratory ability and epithelial‐mesenchymal transition of ultraviolet‐induced SGC cells.^8^ Collectively, our study findings are in conformity with these study outcomes.

Next, for the purpose of translating the mechanism of miR‐651‐5p and CALM2 in lung cancer, a series of assay were conducted with the results implying that miR‐651‐5p overexpression or CALM2 interference repressed the proliferative, migratory, and invasive behaviors of lung cancer cells and promoted apoptosis. As supported by an innovative study, it is reported that CALM2 expression is elevated and has a positive relation to the poor prognosis of GC patients. Furthermore, CALM2 boosts tumor growth and lung metastasis.^14^ As previously demonstrated, CALM2 is linked to poor prognosis in lung cancer patients.^23^ Moreover, it is pointed out that CALM2 silence inhibits proliferation and colony formation of HCC cells via inducing apoptosis.^13^ Lately, a study conducted by Sun et al. has highlighted that CALM2 knockdown can caspase‐dependently promote mitochondrial apoptosis to overcome the resistance to afatinib. Therefore, CALM2 is regarded as a possible target to overcome GC cell resistance to afatinib.^12^ Drawn from a prior study, it is concluded that miR‐338‐3p can directly target CALM2,^16^ which is indicative of the potential interplay between miR‐651‐5p and CALM2. In this research, we have explored the targeting relationship between miR‐651‐5p and CALM2. We found that miR‐651‐5p possessed a target binding site with CALM2. Furthermore, miR‐651‐5p targeted and negatively regulated the expression of CALM2. miRNAs mainly degrade mRNAs by complementary binding to the 3'‐UTR end of their target mRNAs or inhibit the translation of mRNAs, thereby blocking gene expression. When miR‐651‐5p mimic was cotransfected with oe‐CALM2, miR‐651‐5p mimic inhibited CALM2 expression and promoted cell apoptosis, but cotransfection with oe‐CALM2 increased CALM2 levels in cells and the cell apoptosis was inhibited. Therefore, overexpression of CALM2 partially reversed the apoptosis‐promoting effect of miR‐651‐5p mimic on lung cancer cells, which confirmed that the inhibitory effect of miR‐651‐5p on lung cancer was achieved through negative regulation of CALM2 expression. However, the concrete mechanism of the miR‐651‐5p/CALM2 axis needs further expedition.

In summary, the research demonstrates that miR‐651‐5p impacts lung cancer cell proliferative, migratory, and invasive behaviors by regulating CALM2 expression, which may renew the existed knowledge of miR‐651‐5p/CALM2 axis‐targeted mechanism in lung cancer. However, more concrete studies are supposed to be programmed in a larger cohort.

## AUTHOR CONTRIBUTIONS

Yaoguo Lang contributed to study design; Shidong Xu and Yaoguo Lang contributed to manuscript editing; Xianglong Kong and Benkun Liu contributed to experimental studies; Xiangyuan Jin and Lantao Chen contributed to data analysis. All authors read and approved the final manuscript.

## CONFLICT OF INTEREST STATEMENT

The authors declare no conflicts of interest directly related to the contents of this article.

## ETHICS STATEMENT

The tissue specimens and patients' general data were ratified by the Ethics committee of Harbin Medical University Cancer Hospital (approval number: 20200311), and the patients have signed the written informed consent.

## Supporting information


**Table S1.** Primer sequence for RT‐qPCR.
**Table S2.** Correlation between miR‐651‐5p expression and clinicopathological features of patients with non‐small cell lung cancer.

## Data Availability

The data that support the findings of this study are available from the corresponding author upon reasonable request.
