# Supplementary material for: microRNA‐651‐5p affects the proliferation, migration, and invasion of lung cancer cells by regulating Calmodulin 2 expression
Source: Clin Respir J. 2023 Jul 20;17(8):754–63. doi: 10.1111/crj.13665 (PMC10435939; doi:10.1111/crj.13665)
Supplement: Supplementary file 1 — Table S1. Primer sequence for RT‐qPCR. Table S2. Correlation between miR‐651‐5p expression and clinicopathological features of patients with non‐small cell lung cancer. [file CRJ-17-754-s001.docx]

**Supplementary Table 1** Primer sequence for RT-qPCR

| Gene | Primer sequence (5’-3’) |
| --- | --- |
| miR-651-5p | Forward: CGCAGTTTAGGATAAGCTTG |
|  | Reverse: Universal primer |
| CALM2 | Forward: GGGACTCGTTTGCGATGTTC |
|  | Reverse: TGTACAAAGCTAACCATGCTGC |
| U6 | Forward: TCACTTCCTATCGGATCGGC |
|  | Reverse: CTGTACCGACAAAAACACAAGC |
| GAPDH | Forward: GGAGCGAGATCCCTCCAA AAT |
|  | Reverse: GGCTGTTGTCATACTTCTCATGG |

Note: miR-651-5p, microRNA-651-5p; CALM2, calmodulin 2; GAPDH, glyceraldehyde-3-phosphate dehydrogenase.

**Supplementary Table 2** Correlation between miR-651-5p expression and clinicopathological features of patients with non-small cell lung cancer

| Clinicopathological characteristics | High miR-651-5p expression group (n= 34) | Low miR-651-5p expression group (n = 34) | *P* |
| --- | --- | --- | --- |
| Age (years) |  |  | 0.807 |
| < 60 | 16 | 14 |  |
| ≥ 60 | 18 | 20 |  |
| Gender |  |  | 0.224 |
| Male | 21 | 15 |  |
| Female | 13 | 19 |  |
| Tumor size (cm) |  |  | < 0.001 |
| < 3 | 28 | 8 |  |
| ≥ 3 | 6 | 26 |  |
| Lymph node metastasis |  |  | < 0.001 |
| Yes | 10 | 30 |  |
| No | 24 | 4 |  |
| TNM stage |  |  | < 0.001 |
| Ⅰ + Ⅱ | 25 | 8 |  |
| Ⅲ + Ⅳ | 9 | 26 |  |
